# Long-term treatment outcomes of complicated acute diverticulitis in immunocompromised patients

**DOI:** 10.1007/s00384-024-04753-1

**Published:** 2024-11-04

**Authors:** Jorge Sancho-Muriel, Hanna Cholewa, Mónica Millán, David Quevedo, Eduardo Alvarez, Marta Nieto-Sanchez, Raquel Blasco, Francisco Giner, Maria Jose Gomez, Vicent Primo-Romaguera, Matteo Frasson, Blas Flor-Lorente

**Affiliations:** 1 Department of Coloproctology, Hospital Universitario y Politécnico La Fe, Valencia, Spain; 2 University of Valencia, Valencia, Spain

**Keywords:** Diverticulitis, Diverticulosis, Immunosuppression, Immunocompromised patients, Percutaneous drainage

## Abstract

**Purpose:**

The main aim of this study was to determine the short- and long-term outcomes of the non-operative management of acute left-sided complicated diverticulitis (ALCD) in severely immunocompromised patients (IMS group) and compare them with immunocompetent patients (IC group). The secondary aim was to assess the necessity of an elective surgery following a successful prior non-operative management in the IMS group after a non-operative management of the first episode of ALCD.

**Methods:**

Patients presented with their first episode of ALCD between 2012 and 2018 were retrospectively reviewed. Only severely immunosuppressed patients were considered for the analysis, including the following: long-term oral or intravenous steroid intake, current malignancy undergoing chemotherapy, chronic kidney disease on hemodialysis, or solid organ transplant with immunosuppressive medication. For each group, demographic data, severity of the episode, management decisions (conservative or operative), and short- and long-term outcomes were recorded and compared. A sub-analysis of patients with ALCD associating and abscess (modified Hinchey classification Ib/II) was performed.

**Results:**

A total of 290 patients were included in the study: 50 among the IMS and 240 among the IC group. The rate of emergent surgery was higher in the IMS group (50.0% vs. 22.5%, *p* < 0.001) and was associated with increased morbidity (72.4% vs. 50.0%, *p* = 0.041) and mortality (24.1% vs. 4.3%, *p* = 0.003). The duration of the hospital stay was significantly longer in the IMS group (15 vs. 8 days, *p* < 0.001). The final stoma rate was significantly higher in the IMS group (82.1% vs. 22.9, *p* < 0.001), with a median follow-up of 51.4 months. A total of 141 patients presented ALCD with an abscess; 25 in the IMS and 116 in the IC group. There was a higher rate of surgical intervention among the IMS group as the initial treatment approach (24.0% vs. 5.2%, *p* = 0.002), even though the conservative treatment had a similar rate of success (81.3% vs. 92.0%, *p* = 0.178). The recurrence rate following a non-operative approach was similar (IMS: 31.2% vs. 35.4% in the IC group, *p* = 0.169). Furthermore, 81.2% of non-operatively managed IMS patients (13 out of 16) did not require a surgical intervention at the end of the follow-up, with similar findings in the IC group (78/96, 81.2%, *p* = 0.148).

**Conclusion:**

Medical treatment of immunosuppressed patients during their first ALCD episode associated with an abscess is feasible, with a high success rate and results comparable with the IC group. Moreover, taking into account the readmission rates, the need for emergent surgery of the recurrence, and the perioperative mortality and morbidity in the IMS group, conservative management with no differed scheduled surgery seems to be a safe option in this subgroup of patients.

**Supplementary Information:**

The online version contains supplementary material available at 10.1007/s00384-024-04753-1.

## Introduction

Diverticulosis is a common condition in Western countries, with an estimated prevalence of 50% in patients over 60 years old and up to 70% in a subgroup of patients over 80 years old [1]. Acute diverticulitis is the most common complication occurring in up to 10–20% of patients with diverticulosis [2], although this data may be overestimated [3]. Most cases can be managed non-operatively; however, treatment should be personalized according to the disease severity and also taking into account further prognostic factors such as comorbidities and immunosuppression [4, 5].

The incidence of both diverticulitis [6] and immunosuppression [7] has been increasing over the last few decades. Immunocompromised patients (IMS) are reported to have a higher incidence of diverticulitis, associated with more severe disease [7–10] and an increased risk of morbidity and mortality [7, 11]. They also present with more complex recurrences than the immunocompetent population (IC)[10]. Although some authors have recently reported satisfactory results of non-operative management in uncomplicated diverticulitis in immunocompromised patients [12, 13], it is still unclear how to initially manage more complex cases with an associated abscess and whether to proceed with elective surgery following a successful prior non-operative management in this population [3, 14].

The main purpose of this study was to determine the short- and long-term outcomes of the non-operative management of acute left-sided complicated diverticulitis (ALCD) in severely immunocompromised patients (IMS group) and compare them with immunocompetent patients (IC group). The secondary aim was to assess the necessity of an elective surgery following a successful prior non-operative management in the IMS group after a non-operative management of the first episode of ALCD.

## Material and Methods

We performed a retrospective observational study based on a prospectively collected database of patients admitted to our colorectal unit at a tertiary center between 2012 and 2018 due to ALCD (perforation, abscess, obstruction, or fistula) diagnosed by a computed tomography scan (CT). Patients were included only once; readmissions due to recurrent episodes during the study period were not treated as new cases.

Patients corresponding to the IMS group were identified and selected for the purpose of the analysis if they presented at least one of the following factors: long-term oral or intravenous steroid therapy (> 20 mg prednisone per day or equivalents), current malignancy with chemotherapy treatment, chronic kidney disease on hemodialysis, solid organ transplant with the need for immunosuppressive medication. Patients with other forms of moderate immunodeficiency including diabetes, prior splenectomy, or malnourishment were not considered in this study [16].

All of the patients were treated according to the pre-established unit protocol for diverticulitis. Patients with a frank colonic perforation resulting in diffuse peritonitis (both fecaloid or purulent) underwent an emergent surgical intervention. The surgical technique (colonic resection with an end colostomy or primary anastomosis, with or without a temporal stoma) was decided based on the intraoperative findings, patients’ hemodynamic stability, comorbidities, and surgeons’ preference. Patients with complicated diverticulitis with an abscess (pericolic or pelvic < 4 cm) or extraluminal air were initially managed conservatively with intravenous antibiotics (piperacillin-tazobactam 1 g every 8 h or ciprofloxacin 400 mg every 12 h, associating metronidazole 500 mg every 8 h, if allergic to beta-lactam antibiotics), fluids, pain killers and bowel rest. Patients with large pelvic or pericolic abscess (diameter > 4 cm) were considered for image-guided percutaneous drainage. A control CT scan was performed in patients admitted for initial non-operative treatment who failed to improve in 72 h following admission. Treatment failure was defined as the requirement of surgery during the admission or a recurrent episode within 30 days after hospital discharge. Follow-up was done in the outpatient clinic, including a control colonoscopy.

Demographic data, radiological findings, the severity of the episode (according to the modified Hinchey classification [16] and the World Society of Emergency Surgery classification [17]), management decisions (medical treatment, percutaneous drainage, or emergent surgery), short-term outcomes (success rate of the initial treatment, rate of surgery during the admission, admission duration, morbidity, and mortality), and long-term outcomes analyzed during the follow-up (recurrence and its management) were recorded. Also, specific surgical outcomes as a type of the initial surgery, its morbidity and mortality, and the stoma reversal were also recorded.

The Statistical Package for the Social Sciences (SPSS version 22.0, USA) was used for data management and statistical analysis. Patient’s data including the cause of the immunosuppression, radiological findings, therapeutic measures, and outcomes (recurrence, morbidity, mortality) were recorded.

Descriptive statistics are reported as median (25th–75th percentile) for continuous variables and *n* (%) for categorical variables. Differences between both groups were analyzed using the χ2 test for categorical variables and the no parametric Mann–Whitney U test for continuous variables.

As it was a retrospective study, no informed consent was obtained, but the study protocol was reviewed and accepted by the hospital ethics committee. The study was performed following the STROBE guidelines.

## Results

Two-hundred and ninety (290) patients with ALCD were included for the purpose of the analysis, among them, fifty (50) belonged to the IMS group (causes are detailed in Table 1).

**Table 1:** Characteristics of the immunosuppressed patients (IMS group)

Type of immunosuppression	*N* (%)
Solid organ transplant	16 (32.0)
Renal transplant	12 (24.0)
Lung transplant	4 (8.0)
Malignancy	19 (38.0)
Hematological malignancies	10 (20.0)
Chronic kidney failure on hemodialysis	5 (10.0)
Chronic steroid treatment (giant cell arteritis, Behcet’s disease, myasthenia gravis, Polymyalgia rheumatica, rheumatoid arthritis, chronic obstructive pulmonary disease—COPD)	10 (20.0)

The characteristics of both groups are reported in Table 2. There were statistically significant differences between the groups in terms of age and rates of diabetes and hypertension. The severity of the episode based on the CT findings was higher in the IMS group, in both the modified Hinchey and the WSES classification. The non-operative approach was less frequent in the IMS group (50.0 vs. 77.5%, *p* < 0.001). The duration of the hospital stay was significantly longer in the IMS group (15 vs. 8 days, *p* < 0.001). The median follow-up was 51.4 months. The global survival rate at the end of the follow-up was 34.0% in the IMS group vs. 90.8% in the IC group (*p* < 0.001).

**Table 2:** Demographics of the study population — global and divided by subgroups, immunocompetent (IC group), and immunosuppressed (IMS group) patients

	Total number of patients	IC group	IMS group	*p* value
	290 (100.0)	240 (100.0)	50 (100.0)	
Age (median, p25–p75)	59 (49–71)	57 (47–69)	69 (59–76)	** < 0.001**
Sex (*n*, %)				0.345
Male	151 (52.1)	128 (53.3)	23 (46.0)	
Female	139 (47.9)	112 (46.7)	27 (54.0)	
Arterial hypertension (*n*, %)	121 (41.7)	89 (37.1)	32 (64.0)	** < 0.001**
Diabetes (*n*, %)	40 (13.8)	23 (9.6)	17 (34.0)	** < 0.001**
Obesity (*n*, %)	63 (21.7)	52 (22.3)	11 (22.9)	0.928
Episode severity, modified Hinchey classification (*n*, %)				**0.002**
Hinchey 1B	116 (40.0)	97 (40.4)	19 (38.0)	
Hinchey 2	25 (8.6)	19 (7.9)	6 (12.0)	
Hinchey 3–4	44 (15.2)	29 (12.1)	15 (30.0)	
Episode severity, WSES classification (*n*, %)				**0.001**
1A (pericolic air)	76 (26.2)	72 (30.0)	4 (8.0)	
1B (abscess < = 4 cm)	85 (29.3)	71 (29.6)	14 (28.0)	
2A (abscess > 4 cm)	56 (19.3)	45 (18.8)	11 (22.0)	
2B (free distal air)	11 (3.8)	8 (3.3)	3 (6.0)	
3 (free fluid, no air)	2 (0.7)	0	2 (4.0)	
4 (free fluid with free distal air)	42 (14.5)	29 (12.1)	13 (26.0)	
Obstruction (*n*, %)	18 (6.2)	15 (6.2)	3 (6.0)	
Initial treatment (*n*, %)				** < 0.001**
Conservative	211 (72,8)	186 (77.5)	25 (50.0)	
Percutaneous drainage	25 (8.6)	22 (9.2)	3 (6.0)	
Surgery	79 (27.2)	54 (22.5)	25 (50.0)	
Global survival (*n*, %)	235 (81.0)	218 (90.8)	17 (34.0)	** < 0.001**
Hospital stay, days (median, p25–p75)	8 (5–14)	8 (5–11)	15 (9–32)	** < 0.001**
Follow–up, days (median, p25–p75)	1564 (1012–2512)	1684 (1273–2607)	1317 (67–1831)	** < 0.001**

### Surgical outcomes

The rate of emergent surgery was higher among the IMS group (50.0% vs. 22.5%, *p* < 0.001) and was associated with increased morbidity (72.4% vs. 50.0%, *p* = 0.041) and mortality (24.1% vs. 4.3%, *p* = 0.003) (Table 3). Among the patients operated on, there were no statistically significant differences between the IMS and the IC group as to the severity of the episode of the diverticulitis, neither according to the modified Hinchey classification (p = 0.365) nor the WSES classification (*p* = 0.141).

**Table 3:** Characteristics and management of patients operated on due to acute left-sided diverticulitis, divided by subgroups, immunocompetent (IC group), and immunosuppressed (IMS group) patients

	IC group	IMS group	*p* value
	70 (100.0)	29 (100.0)	
Surgery during admission (*n*, %)			0.149
Emergent surgery	48 (68.6)	24 (82.8)	
Differred surgery	22 (31.4)	5 (17.2)	
Surgery type (*n*, %)			**0.003**
Hartmann procedure	35 (50.0)	25 (86.2)	
Primary anastomosis	33 (47.1)	4 (13.8)	
Lavage	2 (2.9)	0 (0.0)	
Estoma (*n*, %)	42 (60.0)	25 (86.2)	**0.011**
Laparoscopic approach (*n*, %)	7 (10.0)	2 (6.9)	0.776
30-day mortality (*n*, %)	35 (50.0)	21 (72.4)	**0.041**
Perioperative death (*n*, %)	3 (4.3)	7 (24.1)	**0.003**
Stoma reversal (*n*, %)	26 (47.3)	4 (14.8)	**0.004**
Stoma present at the end of follow-up (*n*, %)	16 (22.9)	23 (82.1)	** < 0.001**

Also, the rate of colonic resection with an end colostomy (Hartmann’s procedure) was higher in the IMS group (86.2% vs. 50.0%, *p* = 0.003). At the end of the follow-up, the rate of stoma reversal was far lower among the IMS patients, with a final rate of stoma of 82.1% in the IMS group vs. 22.9% in the IC group, (*p* < 0.001).

### Subgroup of patients with complicated diverticulitis with an abscess

One hundred and forty-one (141) patients presented with diverticulitis complicated with abscess formation, where 25 belonged to the IMS group and 116 to the IC group (Table 4). No statistically significant differences were found between the groups (IMS vs. IMC) regarding neither the abscesses’ location (pericolic vs. pelvic) nor their size. Among the IMS group, there was a higher rate of surgical intervention being the initial treatment approach (24.0% vs. 5.2%, *p* = 0.002), even though the initial medical antibiotic treatment had a similar success rate − 81.3% vs. 92.0%, *p* = 0.178. These results did not vary while considering the size of the abscess.

**Table 4:** Characteristics and management of the abscesses’, divided by subgroups, immunocompetent (IC group), and immunosuppressed (IMS group) patients

	IC group	IMS group	*p* value
	116 (100.0)	25 (100.0)	
Location (*n*, %)			0.366
Pericolic	97 (83.6)	19 (76.0)	
Pelvic	19 (16.4)	6 (24.0)	
Abscess size (*n*, %)			
> 3 cm	67 (57.8)	18 (72.0)	0.187
> 4 cm	49 (42.2)	13 (52.0)	0.373
> 5 cm	37 (31.9)	9 (36.0)	0.691
Mean (cm) IC 95%	40.0 (35.5–44.5)	46.7 (35.0–58.5)	0.207
Initial treatment (*n*, %)			**0.002**
Conservative	110 (94.8)	19 (76.0)	
Percutaneous drainage	22 (19.0)	3 (12.0)	
Surgery	6 (5.2)	6 (24.0)	
Medical treatment success (*n*, %)	81 (92.0)	13 (81.3)	0.178
> 3 cm	34 (85.0)	7 (70.0)	0.269
> 4 cm	18 (78.3)	4 (66.7)	0.554
> 5 cm	8 (61.5)	3 (75.0)	0.622
Surgery during the admission (*n*, %)			**0.035**
No	96 (82.8)	16 (64.0)	
Yes	20 (17.2)	9 (36.0)	
Emergent	6 (5.2)	6 (24.0)	
Differred	14 (12.1)	3 (12.0)	
Recurrence (*n*, %)	34 (35.4)	5 (31.2)	0.169
Surgery of the recurrence (*n*, %)	5 (14.7)	2 (40.0)	0.169
Elective surgery (*n*, %)	14 (12.1)	1 (4.0)	0.235
Surgery free survival (*n*, %)	78 (67.2)(81.2)	13 (52.0)(81.2)	0.148

Analyzing the recurrence rate, no differences were found between both groups following an episode of ALCD complicated with an abscess initially managed non-operatively (IMS 31.2% vs. 35.4% in the IC group, *p* = 0.169). Likewise, no differences were found in the need for emergent surgery during the recurrent episode. Furthermore, 81.2% of non-operatively managed IMS patients (13 out of 16) did not require a surgical intervention at the end of the follow-up, with similar findings in the IC group (78/96, 81.2%, *p* = 0.148).

### Type of immunosuppression

We aimed to compare the results between the different subtypes of immunocompromised patients (3 subgroups: patients with either active oncological treatment, solid organ transplant, steroid treatment). No significant differences were found between groups in the severity of the episodes, the initial treatment applied, or short-and long-term results.

## Discussion

The present study shows an elevated success rate of non-operative treatment among immunosuppressed patients with an episode of complicated acute diverticulitis associated with a pericolic or pelvic abscess (81.3% among the IMS groups vs. 92.0% among the IC group, *p* = 0.178).

Recently, there have been publications on satisfactory results of medical treatment of non-complicated left-sided diverticulitis in immunocompromised patients [12, 13]. However, we are not yet in possession of results on ALCD complicated with an abscess (modified HincheyIb/II) in this subgroup of patients. The overall success rate of medical treatment in immunocompetent patients exceeds 80% in current literature [18, 19], which is why it remains the treatment of choice according to different guidelines [4, 5, 17]. These guidelines however present no recommendations on management in immunosuppressed patients.

Hartmann’s procedure was the most common approach among the IMS group (86.2%), comparable with other published series: 65.2% [10]; 79.2% [20];91.4% [9]. Hartmann’s procedure was also the most common intervention among the IC group. Even though the current recommendations tend to encourage primary anastomosis with or without temporary diverting stoma in hemodynamically stable patients with no significant comorbidities [4, 17], in the majority of published series, a colonic resection with an end colostomy continues to be the most common procedure, with higher rates than in our study [21, 22].

The current study shows high rates of morbidity (72.4%) and early mortality (24.1%) among the IMS group, yet tantamount to the current literature — morbidity of 70 [10]–81.8% [21] and mortality of 19.2 [10]–33% [7].

In a recent systematic review of 1599 immunocompromised patients submitted to emergency surgery, the rate of mortality was far superior in the IMS group (RR 1.9, *p* < 0.001) [23]. The same review analyzed the results of elective surgery encountering greater morbidity among the IMS patients (RR 2.18 *p* = 0.04), without differences in the postoperative mortality. A retrospective multicentric review of Al-Khamis et al. [9] included 736 immunocompromised patients and 21,980 immunocompetent and found no morbidity differences between a scheduled sigmoidectomy (OR 1.45) and Hartmann’s procedure (29.2% versus 10.5%, *p* < 0.001).

The high rate of definitive stoma in the IMS group is an important factor to consider (IMS 82.1% vs. IC 29.9%, *p* < 0.001, at the end of the follow-up), which underlines the low rate of stoma reversal in this group of patients, partially due to high surgical risk even in scheduled surgery.

On the other hand, the IMS patients have always been considered a high-risk group for complex recurrences, regardless of the initial staging of diverticulitis [6, 9, 24] and therefore for emergent surgery of the recurrence [5, 24]. Klarenbeek B et al. [24] describe a fivefold higher rate of perforation in the recurrent episode in comparison with the IC patients (36% vs. 7%, *p* = 0.002). On the contrary, other, more recent series present us with lower recurrence rates (12–27.8%) [7, 25, 26]. This is why multiple guidelines recommend submitting the IMS patients to scheduled surgery once they have recovered from the initial episode, regardless of the initial staging [5, 17, 27]. However, recent studies questioned this indication in cases of non-complicated diverticulitis [7, 12], which led to modifications in the recent ASCRS guideline for this subgroup of patients [4], providing a change in comparison to its previous edition [27] and in comparison to other guidelines [5, 17].

Biondo et al. [7] in a retrospective study comparing 107 IMS vs. 657 IC patients (including non-complicated and complicated episodes of diverticulitis) found no differences in the recurrence rates (21.5% IMS vs. 20.5%, respectively, *p* = 0.82), rate of emergent surgery of the recurrence (17.4% IND vs. 15%, *p* = 0.77), after a mean follow-up of 81.6 months. They did find a higher recurrence rate in the IMS patients with an initial episode of ALCD (46.2%, 12/26 patients). The authors concluded that there was no need for scheduled surgery following a non-complicated episode of diverticulitis in IMS patients.

In the present study, we found no significant differences in the recurrence rate following a ALCD with an abscess formation, with a recurrence of 31.2% in the IMS group vs. 35.4% in the IC group (*p* = 0.698). Also, no differences were found in the rate of emergent surgery of the recurrence (*p* = 0.063). In total, 81.2% (13/16) of the IMS patients following a medically managed ALCD did not need surgery at the end of the follow-up, comparable with the IC group (*p* 0.148). We also did not find differences between both groups while analyzing the subgroup of patients with ALCD with localized pneumoperitoneum, without a formed abscess –84.2% did not need surgery at the end of follow-up. These results may question the need for deferred surgery in this subgroup of patients, contrary to other authors [21, 24, 26, 28, 29] and guidelines [5, 17].

The majority of recent studies analyzing the results of immunosuppressed patients with an episode of acute diverticulitis do not take into account the severity of the episode; most of them include non-complicated episodes [10, 13, 23]. In the present study, only complicated episodes were analyzed. Even though there are many conditions which may affect our immune system with distinct severity, for the purpose of our study, only severely immunocompromised patients were considered (solid organ transplant, active oncological treatment, steroid treatment, end-stage kidney failure on hemodialysis) [15, 29]. Whether or not the specific cause of immunosuppression should influence treatment recommendations remainsunknown [4]. In our study, no differences were found between the different subgroups of immunocompromised patients. As it was a unicentric study and the database began following a new protocol establishment, there were no notable differences in the treatment approach. As limitations, we may state the retrospective analysis and the number of patients included.

Medical treatment of immunosuppressed patients during their first ALCD associated with an abscess is feasible, with a high success rate and results comparable with the IC group. Moreover, taking into account the readmission rates, the need for emergent surgery of the recurrence, and the perioperative mortality and morbidity in the IMS group, conservative management with no differed scheduled surgery seems to be a safe option in this subgroup of patients.

## Supplementary Information

Below is the link to the electronic supplementary material.

Supplementary file1 (PDF 375 KB)

## Data Availability

The data that support the findings of this study are not openly available due to reasons of sensitivity and are available from the corresponding author upon reasonable request.
